# A new strategy for the treatment of advanced ovarian cancer: utilizing nanotechnology to regulate the tumor microenvironment

**DOI:** 10.3389/fimmu.2025.1542326

**Published:** 2025-02-12

**Authors:** Zixuan Xiong, Yichun Huang, Shulong Cao, Xuqun Huang, Haiyuan Zhang

**Affiliations:** ^1^ School of Basic Medicine, Health Science Center, Yangtze University, Jingzhou, China; ^2^ Department of Oncology, The First Affiliated Hospital of Kunming Medical University, Kunming, China; ^3^ Department of Pathology, Songzi People’s Hospital, Jingzhou, China; ^4^ Department of Medical Oncology, Huangshi Central Hospital, Affiliated Hospital of Hubei Polytechnic University, Huangshi, China

**Keywords:** advanced ovarian cancer (AOC), tumor microenvironment (TME), chemo-immunotherapy, drug resistance, nanotechnology

## Abstract

Advanced ovarian cancer (AOC) is prone to recurrence, which can be attributed to drug resistance. Drug resistance may be related to the tumor microenvironment (TME), including the immune and non-immune TME. In the immune TME, the immune effector cells such as dendritic cells (DCs), M1-like tumor-associated macrophages (M1-TAMs), and T cells are inhibited. In contrast, immunosuppressive cells such as M2-like tumor-associated macrophages (M2-TAMs), myeloid-derived suppressor cells (MDSCs), and regulatory T cells (Tregs) are activated. These changes make it difficult to produce immune effects and affect the efficacy of chemo-immunotherapy. In the non-immune TME, mechanisms such as apoptosis inhibition, DNA damage response (DDR), and epithelial-mesenchymal transition (EMT) can promote tumor growth, metastasis, and drug resistance. Despite the challenges posed by the TME in the treatment of AOC, the unique biological advantages of nanoparticles (NPs) make it possible to regulate the TME. NPs can stimulate the immune responses of M1-TAMs, DCs, and T cells while reducing the infiltration of immune suppressive cells such as M2-TAMs and Tregs, thereby regulating the AOC immune TME. In addition, NPs can regulate the non-immune TME by reducing apoptosis in AOC cells, inhibiting homologous recombination (HR) repair, reversing EMT, and achieving the effect of reversing drug resistance. In summary, the application of NPs provides some new venues for clinical treatment in AOC.

## 1 Introduction

Ovarian cancer (OC) is the gynecological tumor with the highest mortality rate (1). Due to its unclear early symptoms and lack of effective screening methods, most patients with OC are already in the advanced stage when seeking medical treatment (2). Advanced ovarian cancer (AOC) often spreads to adjacent or distant tissues and organs through implantation, invasion, metastasis, etc., seriously affecting the life quality of patients and reducing their survival time. Tumor cell reduction surgery combined with chemo-immunotherapy is currently a commonly used treatment for AOC (3), but the therapeutic effect is not ideal. In recent years, with the continuous updates of chemotherapy drugs and immunotherapy regimens, the clinical treatment efficacy of AOC has greatly improved. However, patients with AOC are prone to developing drug resistance to chemotherapy and immunotherapy, which is another clinical challenge (4). Therefore, exploring the drug resistance mechanism of AOC cells and improving the drug sensitivity of tumor cells are urgent challenges that need to be addressed in clinical practice.

Related studies have shown that the tumor microenvironment (TME) may be closely related to the development of AOC and the regulation of drug resistance (5). The TME refers to the internal and external environment in which tumor cells are closely related to tumor occurrence and metastasis. It not only includes the function and metabolism of the tumor tissue, but also is related to the internal environment of the tumor cells themselves. TME includes immune cells, mesenchymal cells, and extracellular matrix (6). The TME is a complex and dynamic ecological environment in AOC. It can be regulated by the immune TME such as immunosuppression, immune evasion, or non-immune TME such as apoptosis inhibition, DNA damage response (DDR), and epithelial-mesenchymal transition (EMT). These factors affect the occurrence and development of AOC and lead to drug resistance in ovarian cancer cells (7, 8). Therefore, it may enhance the sensitivity of tumor cells to chemo-immunotherapy and improve the clinical treatment efficacy of patients with AOC by exploring the mechanism of TME in AOC and screening key intervention targets.

Nanotechnology is a technology that studies the properties and applications of materials with structural sizes ranging from 1 nanometer to 100 nanometers, its ultimate goal is to directly construct products with specific functions using atoms or molecules (9). Many reports have shown that the application of nanotechnology can effectively regulate the TME, thereby enhancing its therapeutic effect on malignant tumors (10). For example, constructed NPs can target drug delivery by enhancing the permeability and retention (EPR) effect, significantly increasing drug accumulation at tumor sites, reducing toxicity to normal tissues, and remodeling the TME in breast cancer (11). In the treatment of lung cancer, poly (lactic-co-glycolic acid) copolymer (PLGA) NPs carry targeted drugs, which can prolong the duration of action of the drugs in the body and improve the efficacy of drugs by controlling the release rate of drugs. In addition, effective tumor suppression is due to the direct elimination of lung cancer cells and TAM, thereby regulating the TME (12). The application of NPs not only improves the therapeutic effect, but also reduces side effects, providing strong support for personalized precision medicine. We have reviewed the mechanisms of the TME and introduced NPs that can regulate the TME in AOC, as well as describing their application value and prospects.

## 2 The pitfalls of AOC: the immune TME

The immune TME of AOC is an important component of TME, which is a complex ecosystem composed of tumor cells, immune cells, extracellular matrix (ECM), and various cytokines. There are various immune active cells (ICCs) and immune suppressive cells in the immune TME. Among them, ICCs include dendritic cells (DCs), T cells, B cells, natural killer cells (NK cells), and tumor associated macrophages (TAMs) (13). ICCs are responsible for clearing foreign substances and playing an effector role in immune responses. Immunosuppressive cells, including regulatory T cells (Tregs) and myeloid derived suppressor cells (MDSCs). Immunosuppressive cells mainly exert their immune negative regulatory effects by inhibiting the function of ICC. The immune cells in the immune TME are regulated by various factors, such as the immune checkpoint protein programmed cell death ligand 1 (PD-L1), which can recognize T cells to indicate programmed cell death 1 (PD-1) receptors and mediate immune escape effects. The characteristics of immune TME in AOC are the high concentration of immunosuppressive cells and low concentration of ICC, which greatly reduces the immune ability of ovarian cancer patients. This not only leads to resistance of ovarian cancer cells to chemo-immunotherapy drugs, but also promotes invasion and metastasis of ovarian cancer cells. Therefore, elucidating the mechanism of immune TME can provide a new treatment approach for reversing drug resistance in ovarian cancer cells and improving the efficacy of chemo-immunotherapy.

### 2.1 Immune checkpoint: PD-L1 inhibits activation of T cells in the immune TME

**Figure 1:**
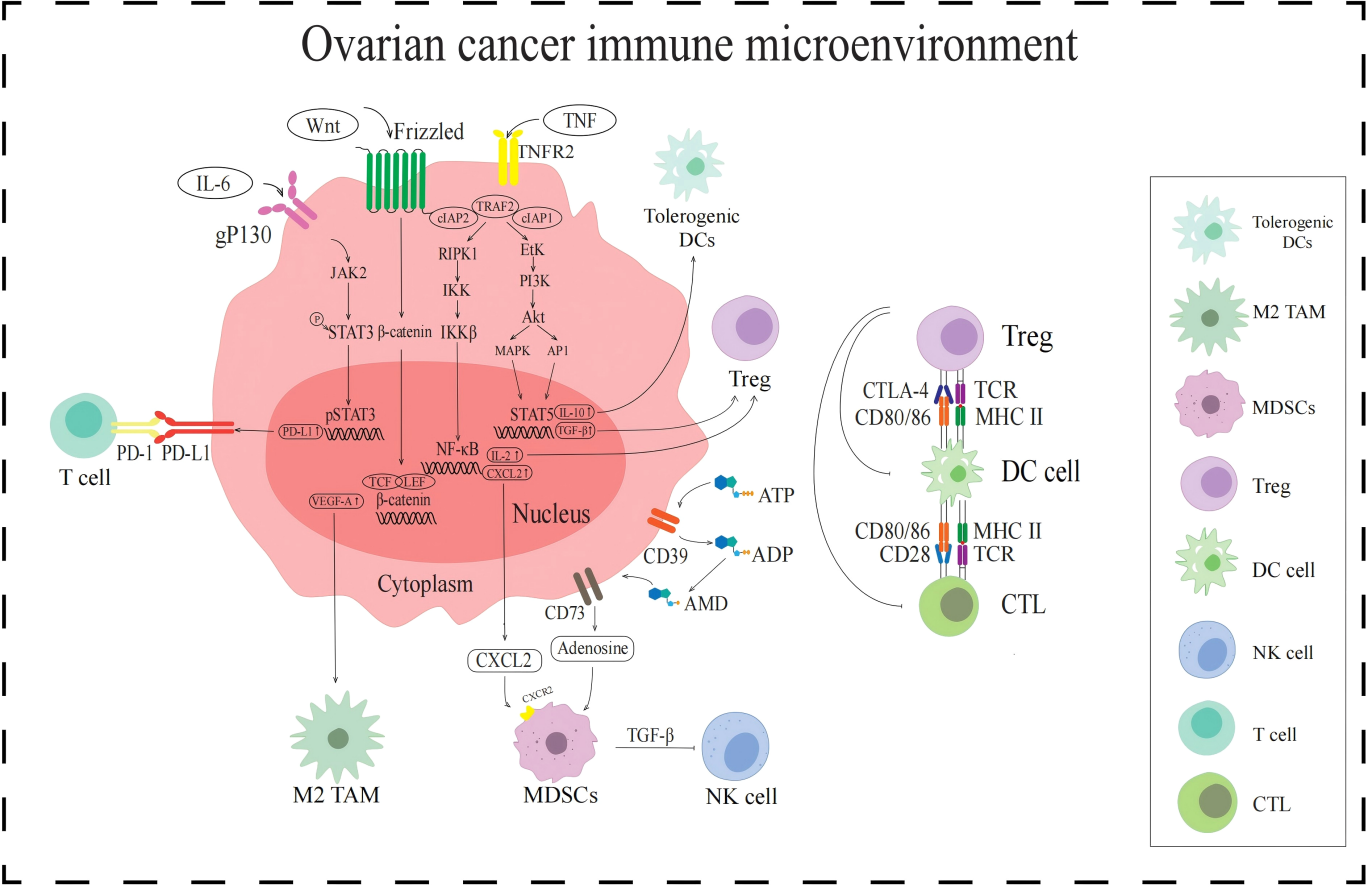
Signaling pathways in the immune TME in AOC. The JAK2/STAT3 signaling pathway upregulates PD-L1 expression and enhances T cell immune escape. WNT/β-catenin signaling pathway increases VEGF-A expression and activates the M2-TAMs. NF-κB signaling pathway and CD39/CD73 activate MDSCs by promoting the secretion of cytokines CXCL2 and adenosine, respectively. MDSCs release TGF-β to inhibit NK cells. Tregs are activated by NF-κB signaling pathway and PI3K/Akt signaling pathway, all of which inhibit DCs and CTL.

PD-L1 is a ligand-protein mainly expressed on the surface of tumor cells and can bind to PD-1. PD-1 is expressed on the surface of T cells and is an important immunosuppressive molecule. PD-L1 in the TME recognizes and binds to PD-1 on the surface of T cells, which can exert functions such as inhibiting T cell proliferation, promoting T cell apoptosis, and reducing cytokine secretion (14). The activated PD-1/PD-L1 signaling pathway can mediate tumor cell evasion of T cell immune response. The transcription factor signaling transducer and activator of transcription 3 (STAT3) plays important regulatory roles in the PD-1/PD-L1 signaling pathway (**Figure 1**), and STAT3 activation is closely related to the regulation of the JAK2/STAT3 signaling pathway. When the cytokine interleukin-6 (IL-6) binds to the tumor cell receptor gp130, it can activate downstream JAK2 protein, which can phosphorylate STAT3 protein. Phosphorylated STAT3 protein forms a dimer and subsequently transfers to the nucleus. The STAT3 protein dimer in the nucleus promotes the transcription and expression of PD-L1 by binding to the promoter region of the PD-L1 gene (15). When the JAK2/STAT3 signaling pathway activates PD-L1, it binds to PD-1 expressed on the surface of T cells, activates the PD-1/PD-L1 signaling pathway, inhibits T cell function, and mediates immunosuppression.

### 2.2 The activity of DCs in the immune TME is inhibited

DCs are antigen-presenting cells (APCs) that play a crucial role in initiating and regulating immune responses. However, the immune TME of AOC not only has a small number of DCs, but also easily leads to functional disorders, which may be related to tumor metabolic regulation. It induces the production of reactive oxygen species (ROS) in response to the high metabolic demands caused by the abnormal proliferation of AOC cells, thereby triggering lipid peroxidation in tumor cells (16). The lipid peroxidation of ovarian cancer cell membranes produces products such as 4-hydroxynonenal (4-HNE) (17). 4-HNE and ROS can act as signaling molecules to transmit oxidative stress signals to DCs, disrupting the endoplasmic reticulum redox homeostasis of DCs, leading to protein misfolding and endoplasmic reticulum stress (ERs) in DCs. The occurrence of ERs can lead to an increase in the synthesis of X-box binding protein 1 (XBP1). XBP1 can regulate lipid metabolism, promote abnormal accumulation of lipids, and lead to fatty acid oxidation (18). The above process may downregulate the ability of DCs to secrete pro-inflammatory cytokines (such as IL-12) and affect the function of DCs.

### 2.3 The role and activation of TAMs in the immune TME

According to their activation status and function, TAMs are divided into different subgroups, namely M1-like tumor-associated macrophages (M1-TAMs) and M2-like tumor-associated macrophages (M2-TAMs) (19). M1-TAMs can release inflammatory substances such as IL-1 and exert anti-tumor effects by promoting inflammatory responses (20). In contrast, M2-TAM secretes anti-inflammatory cytokines such as IL-10 and transforming growth factor β (TGF-β), which are involved in inhibiting immunity and promoting tumor growth (21, 22). However, M1-TAMs are rarely distributed in AOC immune TME, while M2-TAMs are attracted to chemokines regulated by the TME, such as VEGF-A which induced by the WNT/β-catenin signaling pathway. When the WNT ligand binds to the Frizzled receptor on the cell surface, it activates the WNT/β-catenin signaling pathway. In the presence of WNT signaling, degradation of β-catenin is inhibited, allowing it to accumulate in the cytoplasm and transfer to the nucleus. The β-catenin in the nucleus binds to the TCF/LEF family transcription factor and initiates the transcription and expression of the VEGF-A gene. VEGF-A attracts M2-TAMs to the tumor site, resulting in immunosuppressive TME (23) (**Figure 1**).

### 2.4 The activation and function of MDSCs in the immune TME

MDSCs are a type of immune suppressive cells derived from bone marrow, which are precursors of DCs, macrophages, and granulocytes (24). They have the ability to significantly suppress immune cell responses and exhibit significant heterogeneity. The activation of MDSCs is influenced by a variety of factors, such as the chemokine CXC motif chemokine ligand 2 (CXCL2) and the metabolite adenosine (**Figure 1**). CXCL2 is regulated by the NF-κB signaling pathway (25). The NF-κB signaling pathway is a complex and intricate system, with key components including receptors, receptor proximal signaling adaptor proteins, the inhibitor protein family of NF-κB dimers (IκB), IκB kinase (IKK) complexes, and NF-κB dimers. When the NF-κB signaling pathway is activated, IKK plays a key role, and its activation leads to phosphorylation and ubiquitination of IκB protein, which in turn leads to its degradation. With the degradation of IκB protein, NF-κB dimer is released and activated. These activated NF-κB dimers are transferred to the nucleus and can act as transcription factors to bind to the promoter region of the CXCL2 gene, promoting its transcription and expression. CXCL2 activates and recruits MDSCs into the immune TME by binding to CXCR2 on the surface of MDSCs. In addition, the metabolite adenosine is also involved in the activation of MDSCs. Adenosine is produced by ATP molecules through a series of enzymatic reactions. Adenosine activates MDSCs by specifically binding to A2A receptors on the surface of MDSCs, inhibiting immune responses in TME and promoting tumor immune escape (26).

### 2.5 The recruitment of Tregs in the immune TME

Tregs are subpopulations of T cells that are immunosuppressive. When Tregs accumulate in the immune TME, they transmit inhibitory signals by expressing cytotoxic T lymphocyte-associated protein-4 (CTLA-4), thereby preventing DCs from presenting tumor antigens to CTLs, prompting immunosuppression. The activation of Tregs in the immune TME of AOC is influenced by the TNF-TNFR2 axis (27) (**Figure 1**). TNF recognizes and binds to TNFR2 on the surface of tumor cells, and activated TNFR2 acts on and activates downstream receptor-interacting protein kinase 1 (RIPK1). Activated RIPK1 is rapidly ubiquitinated and degraded by the TRAF2-cIAP1-cIAP2 complex, thereby maintaining the signal homeostasis of the TNF-TNFR2 axis. Activated RIPK1 promotes the activation of IKK complexes by phosphorylating IKK subunits. The activation of IKK can lead to the degradation of IKB, resulting in the activation of NF-κB dimer and its entry into the nucleus, regulating the expression of IL-2. And cytokine IL-2 can activate Treg by binding to IL-2R on the surface of Treg. In addition, activated TNFR2 can also act on and activate downstream erythroblast transformation-specific tyrosine kinase (ETK), thereby activating the PI3K/AKT signaling pathway mediated by ETK. ETK activates PI3K to convert phosphatidylinositol diphosphate (PIP2) into phosphatidylinositol triphosphate (PIP3), and then activates downstream protein AKT. The PI3K/Akt pathway mediates phosphorylation activation of STAT5 through a cascade effect. Phosphorylated STAT5 enters the nucleus and recognizes and binds to the regulatory sequence of TGF-β gene, enhancing the expression and secretion of TGF-β. TGF-β in the TME can stimulate Tregs to secrete immunosuppressive cytokines, further exerting their immunosuppressive function.

## 3 The pitfalls of AOC tumor: the non-immune TME

There are some components in the TME of AOC that are not directly related to immune regulation, but can regulate tumor cell growth, metastasis, and drug resistance. We classify it as the non-immune TME.

### 3.1 Non-immune TME regulates proliferation and migration of AOC cells

**Figure 2:**
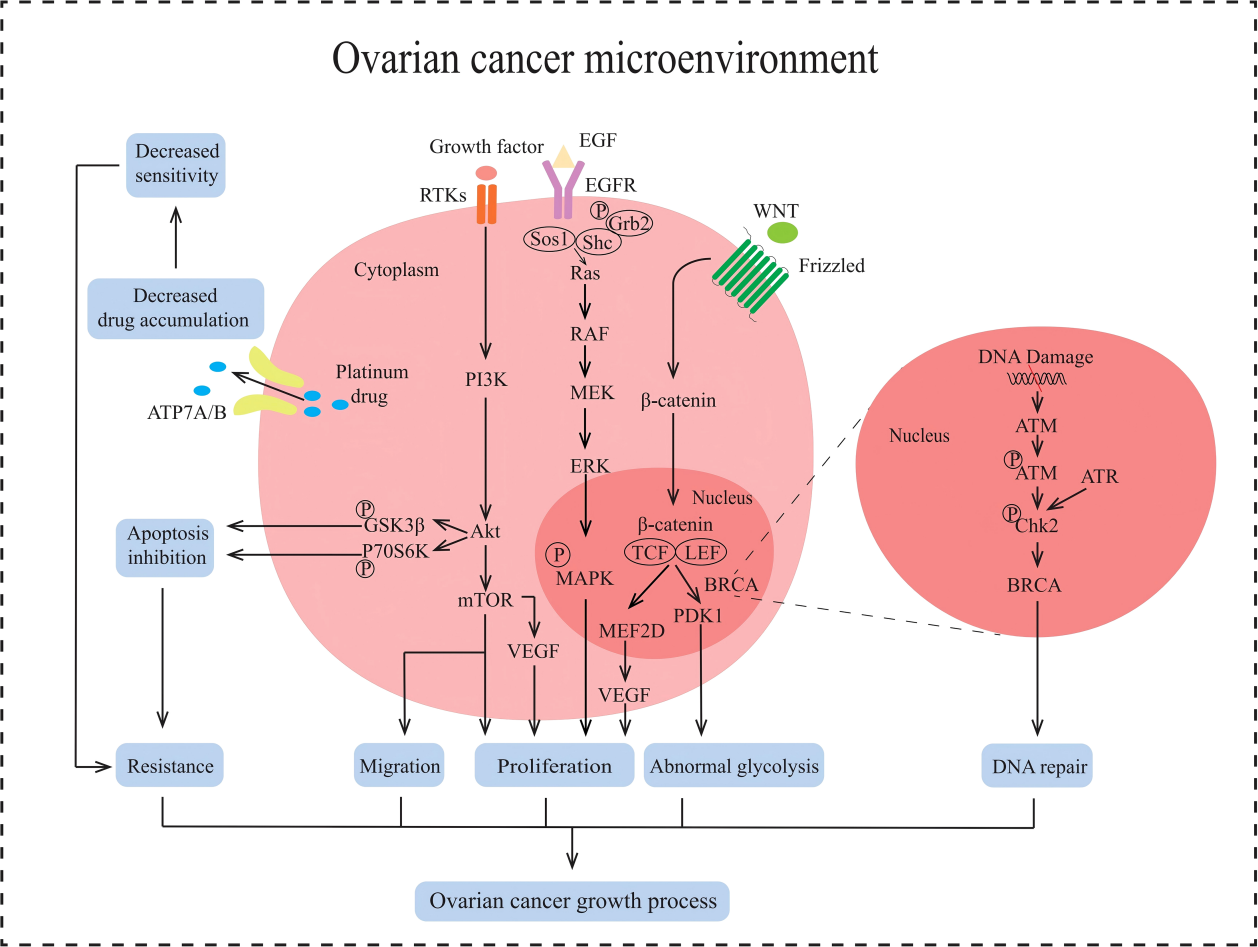
Multiple signaling pathways in the non-immune TME in AOC. ATP7A/B causes the efflux of platinum drugs. PI3K/AKT/mTOR signaling pathway promotes tumor proliferation, induces EMT and enhances tumor metastasis, inhibits apoptosis and protects against autophagy in AOC. RAS/RAF/MAPK signaling pathway promotes proliferation in tumor. Wnt/β-catenin signaling pathway stimulates cell proliferation and abnormal glycolysis in AOC. BRCA cause DNA damage repair.

In non-immune TME, tumor cell growth is mainly regulated by the PI3K/AKT/mTOR signaling pathway, RAS/RAF/MAPK signaling pathway, and WNT/β-catenin signaling pathway (**Figure 2**). Abnormal activation of the PI3K/AKT/mTOR pathway can inhibit tumor cell apoptosis, promote tumor cell growth and proliferation, facilitate tumor cell angiogenesis and metabolism, participate in tumor cell invasion and metastasis (28). The activation of the PI3K/AKT/mTOR pathway is closely related to the regulation of receptor tyrosine kinase (RTK). Growth factors in the non-immune TME recognize and bind to RTK on the surface of tumor cells. Activated RTK exerts protein kinase activity, recruits PI3K, and converts PIP2 into PIP3, thereby activating downstream AKT. Activated AKT regulates protein and lipid synthesis by phosphorylating and activating mTORC1, promoting abnormal proliferation of tumor cells. The RAS/RAF/MAPK signaling pathway is another key pathway for non-immune TME regulation of AOC occurrence and development. This pathway is initiated by the binding and activation of epidermal growth factor (EGF) to the cell surface receptor EGFR. After receptor activation, son of sevenless (Sos) are recruited to the plasma membrane. The function of Sos is to convert the Ras on the inner side of the cell membrane from GDP-Ras to GTP-Ras, thereby activating Ras. Activate Ras recruitment and activate RAF, leading to RAF phosphorylation. Phosphorylated RAF subsequently activates downstream MEK, which in turn activates ERK. Activated ERK is transferred to the nucleus and stimulates phosphorylation of various transcription factors, including myeloid leukemia factor 1 (Mcl-1), cellular oncogene Jun (c-Jun), and cellular oncogene fos (c-fos). These transcription factors promote cell proliferation, survival, and cell cycle progression (29). The WNT/β-catenin signaling pathway also plays an important role in the occurrence and development of ovarian cancer. When the WNT ligand recognizes and binds to Frizzled receptors on the cell surface, it facilitates the formation of a WNT protein-Frizzled receptor-LRP5/6 trimer. This trimeric complex activates the intracellular Dvl protein, destabilizing the APC-Axin-GSK-3β degradation complex. As a result, β-catenin phosphorylation and subsequent degradation are inhibited, leading to an increase of β-catenin in the cytoplasm, which then translocates into the nucleus. The β-catenin entering the nucleus binds to the TCF/LEF transcription factor family, initiating the transcription and expression of downstream target genes such as MEF2D. MEF2D can enhance the expression of the VEGF gene in tumor cells, thereby promoting angiogenesis and inducing abnormal proliferation of tumor cells (30, 31). In summary, the non-immune TME can regulate the proliferation and metastasis of ovarian cancer cells by activating the three signaling pathways mentioned above.

EMT is one of the key processes in the development of ovarian cancer and refers to the transformation of epithelial cells into mesenchymal cells (32), giving tumor cells the ability to migrate, invade, and promote tumor metastasis and recurrence. It has been reported that the induction of EMT is associated with the TGF-β signaling pathway (33) (**Figure 2**), which is divided into Smad-dependent and Smad-independent pathways (34). In the Smad-dependent pathway, TGF-β binds to its receptor and activates Smad proteins, leading to the translocation of Smad complexes into the nucleus to regulate the downregulation of E-cadherin expression and upregulation of mesenchymal markers such as N-cadherin and Vimentin in ovarian cancer epithelial cells. Furthermore, within the Smad-independent pathway, TGF-β can activate additional signaling pathways such as MAPK and PI3K/AKT, which collectively regulate the expression of transcription factors including Snail, Twist, and ZEB1. This regulation leads to the inhibition of E-cadherin expression and facilitates the process of EMT. Non-immune TME induces EMT in AOC cells and promotes tumor cell migration.

### 3.2 Drug resistance induced by the non-immune TME

The occurrence of drug resistance in AOC is a complex process influenced by multiple factors, including the regulation of the immune TME mentioned previously. Currently, some studies have suggested that the non-immune TME can also regulate drug resistance in AOC cells. We have summarized some non-immune TME related factors that lead to drug resistance in AOC, including abnormal glycolysis, inhibition of tumor cell apoptosis, reduction of drug accumulation, and DDR.

#### 3.2.1 Non-immune TME in AOC regulates abnormal glycolysis in tumor cells

Cancer cells preferentially produce energy through glycolysis even when oxygen is abundant. This process, known as the Warburg effect, enables the rapid production of ATP and metabolic intermediates, supporting the rapid proliferation and survival of cancer cells (35). Some miRNAs in the non-immune TME of AOC play an important role in the Warburg effect, such as miR-1180. MiR-1180 inhibits the translation of SFRP1 mRNA by targeting its 3’UTR, thereby reducing extracellular SFRP1 protein levels. As a negative regulator of the WNT/β-catenin pathway, SFRP1 typically inhibits the pathway by binding to WNT ligands or Frizzled receptors (**Figure 2**). Consequently, the reduction of SFRP1 leads to overactivation of the WNT/β-catenin signaling pathway and upregulates the expression of the key glycolytic enzyme PDK1, which promotes glycolysis by inhibiting pyruvate dehydrogenase (PDH) activity (36). The abnormal upregulation of glycolysis may be one of the reasons why non-immune TME regulates drug resistance in AOC cells.

#### 3.2.2 Non-immune TME in AOC regulates apoptosis inhibition in tumor cells

Apoptosis is a programmed cell death that is regulated by intracellular gene expression and signaling pathways, and is an important mechanism for the body to eliminate damaged, infected, or tumor cells. However, in order to promote their survival and proliferation, tumor cells inhibit apoptosis through various mechanisms, such as the regulation of aberrant miRNAs in the non-immune TME of AOC (**Figure 2**). The increased formation of miRNAs, such as miR-214, miR-93 and miR-223, inhibits tumor suppressor gene PTEN (37–39), activates PI3K/AKT signaling pathway, stimulates phosphorylation of downstream glycogen synthase kinase 3b (GSK3b) and p70 ribosomal protein S6 kinase (p70S6K), inhibits the activity of apoptosis-related proteins such as BAD (38, 40). Therefore, the inhibition of apoptosis induced by the non-immune TME of AOC can lead to drug resistance in tumor cells.

#### 3.2.3 Non-immune TME in AOC can regulate the decrease of drug accumulation in tumor cells

Platinum-based drugs are first-line chemotherapy drugs for AOC adjuvant therapy, but due to their long usage cycle, ovarian cancer cells are prone to develop drug resistance to platinum-based drugs. The molecular mechanism of drug resistance in ovarian cancer cells may be related to copper efflux transporters, including copper transporter 1 (CTR1), ATP7A, and ATP7B (41) (**Figure 2**). CTR1, as the main transporter protein of platinum drugs, has been shown to affect the intracellular content of platinum drugs at its expression level. It can reduce the expression of CTR1 protein in cancer cells due to inflammatory factors produced in the non-immune TME of AOC, leading to a decrease in the effective content of platinum-based chemotherapeutic agents within the cells and inducing drug resistance (42). In addition, ATP7A and ATP7B are also involved in regulating the transport of platinum-based drugs within tumor cells (43). Given that tumor cells are metabolized through aerobic fermentation, ROS present in the non-immune TME of AOC upregulates the expression of ATP7A and ATP7B by activating the Nrf2 signaling pathway. ATP7A can isolate platinum-based drugs into cell vesicles, reducing their contact with tumor cell DNA. ATP7B promotes the transport of platinum-based drugs to the extracellular space. In summary, copper flux transporters generate drug resistance by reducing the intracellular levels of chemotherapy drugs in tumor cells.

#### 3.2.4 Non-immune TME in AOC regulates DDR in tumor cells

DDR is one of the fundamental physiological mechanisms in biology, which is a highly conserved mechanism for cells to resist DNA damage induced by external and internal factors. But ovarian cancer cells can repair the damage to the tumor cell genome caused by platinum-based chemotherapy drugs by inducing excessive activation of DDR. ROS produced by aerobic fermentation of ovarian cancer cells can activate ATM and ATR in tumor cells, leading to phosphorylation of downstream Chk2 kinase. Chk2 kinase can activate downstream BRCA proteins, thereby regulating the DDR effect and leading to resistance of ovarian cancer cells to platinum-based drugs (44) (**Figure 2**).

## 4 The strategy for pitfall in AOC: application of nanotechnology in the regulation of the immune TME

Although the presence of an immune TME poses a significant challenge in the treatment of AOC, the inherent targeting, multifunctionality, and pharmacokinetic advantages of NPs allow precise regulation of the immune TME. The role of nanotechnology in AOC chemo-immunotherapy has attracted widespread attention. We have summarized and reviewed the reported applications and research, listing various NP designs, including their compositions and corresponding signaling pathways (**Table 1**).

**Table 1:** Application of NPs in the immune TME of AOC.

Categories	NPs	Material	Medicine	Function	Pathway	Ref.
TAMs	UBR5-silencing nanoparticles	MSV	Ubr5 siRNA polyplex	Disrupted paracrine regulation of TAMs infiltration	Wnt/β-catenin	(45)
TAMs	VSSP	NAcGM3 and OMV	VSSP	Polarization; Altered surface markers, cytokine secretion	\	(46)
TAMs	RSQ-loaded liposomes	Liposomes	RSQ	TAM activation; Induced Th1 response	NF-κB	(47)
TAMs	HA-PEI-miR- 125b	HA-PEI	miR-125b	Repolarized TAMs; Reduced VEGF	\	(48)
TAMs	miR497/TP- HENPs	Exosomes, liposomes	TP, miR497	Polarization; Increased TNF-α	\	(49)
DCs	KT-NE	nanoemulsion	KIRA6-loaded α-Tocopherol	Reduced lipid abundance in TIDCs; Restored DCs-dependent immunity	\	(50)
DCs	FCM-NPs	PLGA	CpG-ODN and FCs Membrane	Activated CTLs, IFN-γ and so on	\	(51)
DCs	IRO@FA NPs	PLGA	PFH cores surrounded by IR780	Increased mature DCs and T cells	\	(52)
DCs	Fe3O4-ICG@ IRM	Fe3O4 NPs	IRM	Increased tumor antigens	\	(53)
DCs	CbP/siPD-L1@Dig	CuS NPs	Dig and siPD-L1	Apoptosis; Immunogenicity; Reduced PD-L1	\	(54)
T cells	TNFR2 antibody NPs	\	TNFR2 antibodies	Reduced TNFR2	NF-κB;PI3K/Akt	(27)
T cells	PbAE-mRNA polyplexes	PbAE	IVT mRNA	Triggered CD3 clustering; Activated T cells	\	(55)
T cells	Exo-miR-155-5p	Exosome	miR-155-5p	Downregulated PD-L1 and activated CD8+ T cell function	miR-155-5p/PD-L1	(56)

### 4.1 Regulation of TAMs in the immune TME of AOC

**Figure 3:**
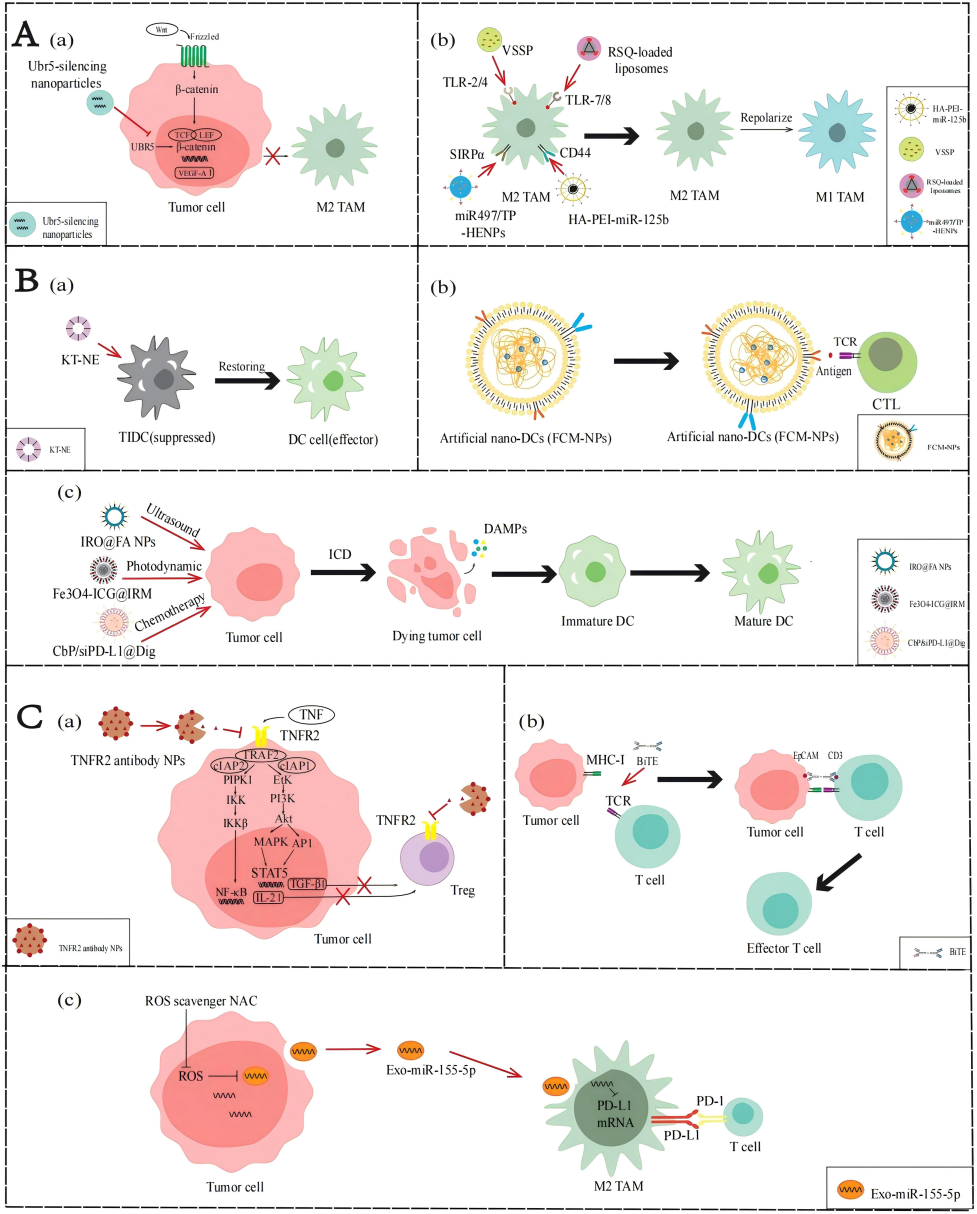
Applications of NPs to reverse the immune TME in AOC. **(A)** Application of NPs targeting TAMs. (a) Ubr5-silencing NPs inhibit the WNT/β-catenin signaling pathway to stimulate M2-TAMs activation. (b) VSSP, RSQ-loaded liposomes, HA-PEI-miR-125b and miR497/TP-HENPs promote TAMs polarization. **(B)** Application of NPs targeting DCs. (a) KT-NE can restore DCs to produce antigen presentation. (b) FCM-NPs are used as a synthetic DCs that acts like normal DCs. (c) IRO@FA NPs, Fe3O4-ICG@IRM and CbP/siPD-L1@Dig exert the ICD effect and activate the function of DCs. **(C)** NP applications targeting T cells. (a) TNFR2 antibody NPs inhibit the NF-κB signaling pathway and PI3K/Akt signal pathway to suppress Tregs activity. (b) BiTE can promote the recognition of T cells and tumor cells. (c) miR-155-5p down-regulate PD-L1 and promote T cells activity.

In the immune TME of AOC, M2-TAMs have tumor-promoting properties and infiltrate abundantly, but M1-TAMs with anti-tumor properties are rarely distributed. Currently, many molecular drugs, such as TLR agonists or miRNAs, can be developed to promote TAM polarization. However, owing to factors such as low bioavailability, poor targeting, and instability *in vivo*, these drugs often fail to achieve the expected effects *in vivo*. However, nanotechnology can be used to convert these drugs into NPs and improve their targeting and utilization, thereby achieving the polarization of TAMs and improving the immune TME of AOC (**Figure 3A**).

#### 4.1.1 NPs terminate the recruitment of M2-TAMs by regulating the immune TME in AOC

As mentioned earlier, activation of the WNT/β-catenin signaling pathway upregulates VEGF-A, mediating the activation and infiltration of M2-TAMs. Therefore, inhibition of the WNT/β-catenin pathway may reduce the infiltration of M2-TAM in the immune TME. Song et al. (45) found that the ubiquitin protein ligase E3 component n-recognin 5 (UBR5), a ubiquitin ligase homologous to the E6AP C-terminal (HECT) domain, can maintain β-catenin activation and upregulate key chemokines and cytokines to promote the recruitment and activation of M2-TAMs. Constructing NPs loaded with UBR5 siRNA can block the WNT/β-catenin signaling pathway and downregulate the VEGF-A gene by targeting and silencing UBR5 expression, thereby reducing the recruitment of M2-TAMs.

#### 4.1.2 NPs repolarize TAMs by regulating the immune TME in AOC

The phenotypic state of TAMs is highly dynamic, allowing them to respond to various factors in the immune TME and switch between M2 and M1 phenotypes, a process known as polarization. Currently, many molecular targeted drugs for TAM polarization have been developed, such as TLR agonists. TLR agonists can bind to TLRs on the surface of TAMs, activate the TLR signaling pathway, induce the expression of M1 markers including CD86, iNOS, and MHC-II in TAMs, and reduce the expression of M2 markers including CD206, CD163, and Arg-1, thereby promoting the transformation of TAMs from M2 phenotype to M1 phenotype. Recently, Khan et al. (46) developed an NPs composed of GM3 ganglioside (NAcGM3) containing N-acetylneuraminic acid and Neisseria meningitidis outer membrane vesicles (OMV) (VSSP) assembled through hydrophobic interactions, which can act as TLR agonists to activate the transformation of M2-TAM to M1-TAM in the immune TME. In their report, the polysaccharide structure of NAcGM3 can be recognized by TLR-2, while the OMV component containing lipopolysaccharide (LPS) activates TLR-4, allowing VSSP to act as TLR-2/4 agonists. VSSPs converted M2-TAMs to M1-TAMs by downregulating the expression of M2-TAM marker CD206, and increasing the secretion of pro-inflammatory cytokines such as TNF-α and IL-1β, reversing immune suppressive cell function and enhancing immune activity in the immune TME. Kang et al. (47) prepared resiquimod (RSQ) into specific NPs and precisely delivered them into tumor cells. RSQ is a TLR-7/8 agonist with immune stimulatory ability, which can promote the conversion of M2-TAM to M1-TAM, thereby improving the immune TME and enhancing anti-tumor immune response.

Parayath et al. (48) found that some miRNAs affect the TAM phenotype, with miR-125b having the highest repolarization efficiency. Therefore, they encapsulated miR-125b with hyaluronic acid- poly(ethyleneimine) (HA-PEI) NPs that specifically target TAMs to prepare HA-PEI-miR-125b NPs, thereby inducing phenotypic transformation of TAMs. In ID8 OC model mice administered HA-PEI-miR-125b NPs by intraperitoneal injection, the experimental group showed a significant decrease in M2-TAMs and a significant increase in M1-TAMs. These results demonstrate that the nanotechnology-mediated delivery of miR-125b can effectively repolarize TAMs in immune TME and be used in combination with chemotherapy drugs to enhance anti-tumor effects, providing a new idea for the treatment of ovarian cancer.

### 4.2 Regulation of DCs in the immune TME of AOC

Although DCs have been shown to play a central role in tumor-specific immune responses, the infiltration of DCs in the immune TME of AOC is relatively insufficient. To enhance the function of DCs, a variety of strategies can be adopted: first, to improve the antigen presentation capacity of under functioning DCs; second, prepare artificial DCs to enhance the immune response; finally, immunogenic cell death (ICD) is used to activate the function of DCs (**Figure 3B**).

#### 4.2.1 NPs improve ineffective DCs by regulating the immune TME in AOC

As described above, lipid accumulation and ERs regulated by the immune TME in AOC affect the function of DCs. Restoring the functionality of DCs is a topic worth studying. Lu et al. designed a KT-NE nanoemulsion to target the lipid accumulation caused by abnormal ER and oxidative stress in the TME (50). The main components of KT-NE are KIRA6 and α-tocopherol. KIRA6 is an advanced small molecule IRE1α kinase and RNase inhibitor that reduces the synthesis of FAs through XBP1. α-Tocopherol relieves oxidative stress, blocks the generation of 4-HNE, and is a powerful ROS scavenger. In summary, the construction of NPs can synergistically inhibit the ROS-triggered lipid peroxidation, repair the function of damaged DCs in the immune TME, enhance the efficiency of antigen presentation, activate T cell-mediated immune responses, and improve the effect of chemo-immunotherapy.

#### 4.2.2 Artificial NPs replace DCs in the immune TME

Even without effective DC penetration, we can use biomimetic nanomaterials to manufacture artificial DC NPs. Zhang et al. (51) prepared FCM-NPs, artificial biomimetic nanoDCs that can exhibit antigen presentation and immunostimulatory effects in the immune TME. The preparation of FCM-NPs involves first fusing ovarian cancer cells with DCs in mouse bone marrow, using the polyethylene glycol (PEG) method to generate fused cells (FC), preserving the immunogenicity of tumor cells and the antigen presentation function of DCs, and extracting the cell membrane of FC. Then select PLGA-NPs encapsulate the extracted fusion cell membrane and immune modulator CpG-ODN, and finally make FCM-NPs. Therefore, FCM-NPs have the antigen presentation ability of DCs, increases the expression levels of co-stimulatory molecules such as CD80 and CD86, and stimulates immature T lymphocytes to produce a large number of tumor-specific cytotoxic CD8+T lymphocytes. According to the experimental results, FCM-NPs exhibit strong immune activation and significant anti-tumor effects in both *in vitro* and *in vivo* experiments, and have good biocompatibility. This shows that constructing NPs to simulate the antigen presentation and immune activation functions of DCs can effectively enhance the activation of CTLs, thereby reshaping the immune TME, and improving the effect of chemo-immunotherapy.

#### 4.2.3 NPs activate DCs by inducing ICD in immune TME of AOC

When tumor cells die under external stimuli, the process by which they can transition from non-immunogenic to immunogenic and mediate the body’s anti-tumor immune response is called ICD. When tumor cells develop into ICD, they produce a series of signaling molecules called damage-associated molecular patterns (DAMPs), which mainly include calreticulin (CRT), high mobility protein 1 (HMGB1), ATP molecules, and heat shock proteins (HSP70 and HSP90). These molecules can bind to pattern recognition receptors (PRRs) on the surface of DCs, thereby attracting DCs to migrate to the tumor site and enhancing the recognition and attack of immune cells on the tumor. Ling et al. (54) designed a nanoscale coordination polymer particles (NCP) with a core encapsulating the chemotherapeutic drugs carboplatin and siPD-L1 and a shell incorporating digoxin(Dig), called CbP/siPD-L1@Dig. Once these NCPs are taken up and ruptured intracellularly, Dig, an ICD inducer, induces immunogenicity, carboplatin induces apoptosis, and siPD-L1 can knock down PD-L1 expression to reduce immunosuppression. The results showed that cells treated with CbP/siPD-L1@Dig significantly increased the release of DAMPs such as ATP and HMGB1, activated DCs, promoted their maturation and antigen presentation ability, and regulated the immune TME to facilitate chemo-immunotherapy. Similarly, Zheng et al. (52) designed a sonodynamic therapy (SDT) nanoplatform to induce ICD in ovarian cancer cells. They encapsulated the photosensitizer IR780 with PLGA and attached FA for improved targeting, then added perfluorohexane (PFH), an oxygen-carrying material, and named it IRO@FA NPs. Experiments showed that the DAMPs changed significantly and the expression levels of IL-6 were significantly elevated in ovarian cancer cells treated with IRO@FA NPs, suggesting that ICDs are induced in tumor cells and activate DCs, initiating an immune response. Therefore, the construction of NPs to induce ICD can activate the function of DCs in the immune TME, promote antigen presentation, and improve the body’s immune response, thereby improving the effect of chemo-immunotherapy.

### 4.3 Regulation of T cells in the non-immune TME in AOC

T cells play a crucial role in enhancing the immune response of patients. There are often three ways to activate the positive regulatory role of T cells in AOC immune TME: one is to terminate the recruitment of Tregs, the other is to increase the infiltration of effector T cells, and the third is to increase the role of exosomes (**Figure 3C**).

#### 4.3.1 NPs terminate Treg recruitment by regulating the immune TME in AOC

As previously described, the NF-κB or PI3K/Akt signaling pathways in the immune TME of AOC can promote Treg infiltration by regulating relevant cytokines. If these aforementioned pathways are blocked, Treg activation in TME may be downregulated. Al-Hatamleh et al. (27) proposed the following hypothesis. They suggest that blocking the TNF-TNFR2 axis by NPs containing TNFR2 antibodies can affect the activation of NF-κB or PI3K/Akt signaling pathways, downregulating Treg-associated cytokines such as TGF-β and IL-2, thereby reducing the activation and distribution of Tregs.

#### 4.3.2 NPs increase infiltration of effector T cells by regulating the immune TME in AOC

Owing to the lack of T cell infiltration in the immune TME of ovarian cancer, recruiting T cells from other locations to achieve chemo-immunotherapy could be a promising strategy, and bispecific T cell engagers (BiTEs) can achieve it. BiTE is an artificial antibody with two antigen-binding sites, one binding to the CD3 molecule on the T cell and the other binding to a specific antigen on the tumor cell, thereby bringing the T cells and the tumor cells together and activating the killing function of the T cells (57). However, the imprecise targeting and non-tumor toxicity of BiTE in TME make it less effective against solid tumors. Hao et al. (55) designed targeted nanocarriers that can deliver mRNA encoding BiTE, which is transcribed *in vitro*, to bone marrow cells that can target immune TME. The result indicated that when encountering BiTEs-bound tumor cells, T cells can be activated, secrete the pro-inflammatory cytokine IFN-γ, and thus effectively kill ovarian cancer cells.

#### 4.3.3 Exosomes reduce immune checkpoint suppression by regulating the immune TME in AOC

Exosomes are membrane vacuoles released into the extracellular matrix after the fusion of multivesicular bodies (MVBs) with the cell membrane, which are important mediators of intercellular substances and signal transduction. They affect the biological functions of recipient cells by carrying and transmitting bioactive molecules, such as exosome delivery of miR-155-5p inhibiting the inactivation of T cells and the infiltration of TAMs by targeting PD-L1. However, the ROS produced by the indefinite proliferation of AOC cells can inhibit the expression of miR-155-5p in exosomes. Li et al. (56) found that miR-155-5p levels in tumor cell exosomes increased after neutralizing ROS by using N-acetylcysteine (NAC). This treatment not only increases the proportion of CD8+ T cells and decreases T cell apoptosis, but also decreases macrophage migration capacity and tumor spheroid infiltration. This study reveals the important role of ROS and exosomal miR-155-5p in the immune TME of AOC, providing a theoretical basis for the development of new therapeutic strategies in the future.

## 5 The strategy for pitfall in AOC: application of nanotechnology in the regulation of non-immune TME

Although non-immune TME presents many challenges for the treatment of AOC, various NPs offer promising strategies for addressing these challenges. We have summarized and reviewed the reported applications and research, listing various NP designs, including their compositions and corresponding signaling pathways (**Table 2**).

**Table 2:** Application of NPs in the non-immune TME of AOC.

Categories	NPs	Material	Medicine	Function	Targeted point/Pathway	Ref.
Metastasis	GNP	Gold NPs	\	Reversing EMT	MAPK; PI3K/AKT	(58)
Drug resistance	R-Se@MEF2D-siRNA	SeNPs	\	Apoptosis; mitochondrial dysfunction	WNT/MEF2D	(31)
Drug resistance	Sal-AgNPs	AgNPs	salinomycin	Apoptosis; autophagy	\	(59)
Drug resistance	miR497/TP-HENPs	Exosomes,liposomes	TP; miR497	Increased ROS; apoptosis	PI3K/AKT/mTOR	(49)
Drug resistance	MEnZn-CuO NPs	Zn-CuO NPs	\	Inhibited DNA repair	BRCA	(60)
Drug resistance	RGD-CaPO/DOX	RGD	CaPO/DOX	ER stress; calcium overload; mitochondrial dysfunction	\	(61)
Drug resistance	IDEM-DOXO	DOXO	IDEM	Apoptosis	\	(62)

### 5.1 NPs improve the sensitivity of AOC therapy by regulating the non-immune TME

Multiple mechanisms underlie drug resistance caused by non-immune TME in AOC. Many reports have indicated that the application of nanotechnology can effectively regulate non-immune TME, reverse drug resistance and improve the treatment sensitivity. We have summarized some examples of the modulation of the non-immune TME by constructing specific NPs, including the promotion of apoptosis, inhibition of proliferation, prevention of DDR, and reversal of EMT.

#### 5.1.1 NPs promote apoptosis by regulating the non-immune TME in AOC

**Figure 4:**
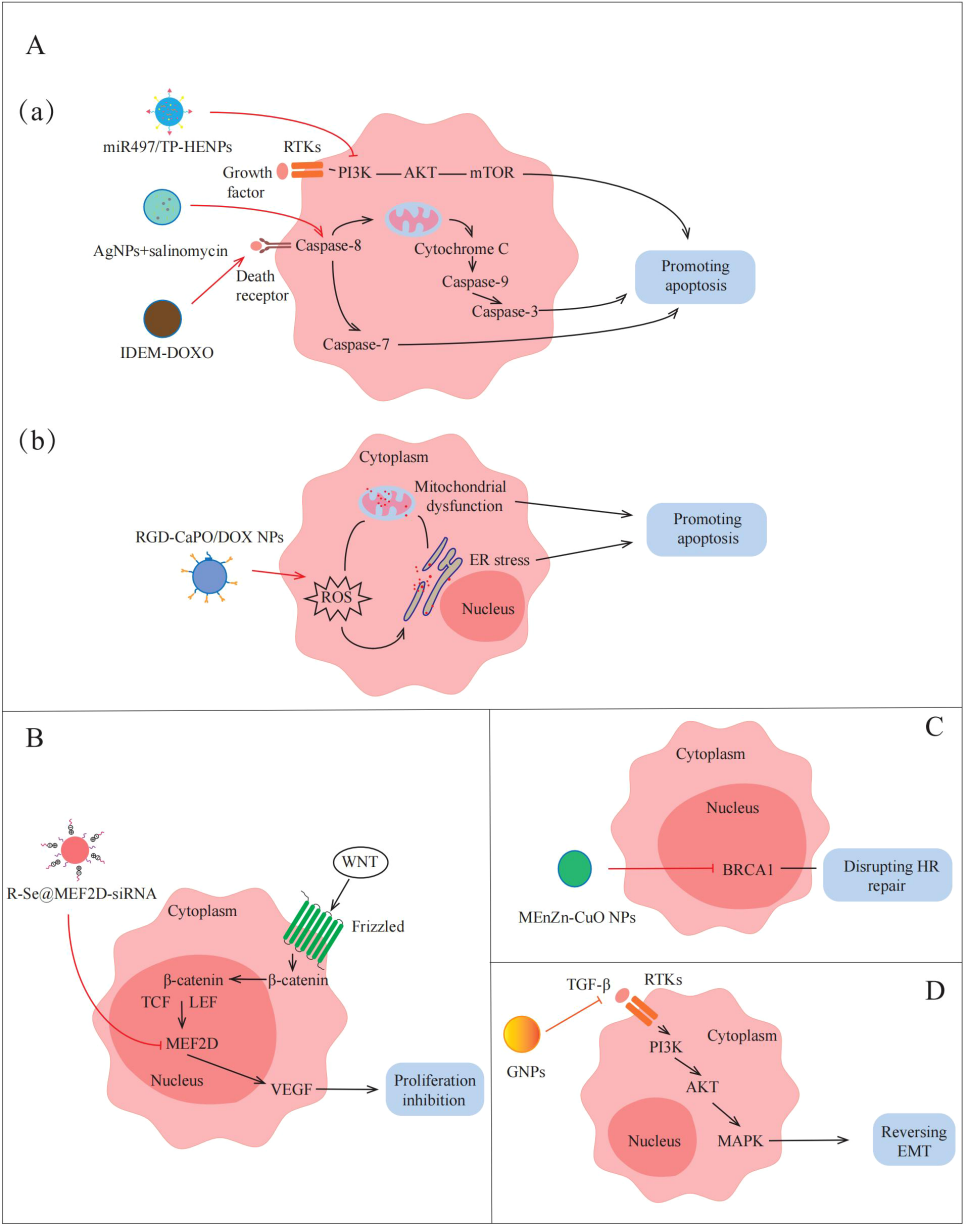
Applications of NPs to reverse the non-immune TME in AOC. NPs destroy tumors by promoting apoptosis, inhibiting proliferation, disrupting HR repair and reversing EMT. **(A)(a)**(a) miR497/TP-HENPs can promote apoptosis by inhibiting PI3K/AKT/mTOR signaling pathway. Sal-AgNPs and IDEM-DOXO promote AOC cell apoptosis by activating Caspase-3-depended signaling pathways. (b) RGD-CaPO/DOX NPs and R-Se@MEF2D-siRNA induce apoptosis directly via increasing ROS preparation, mitochondrial damage and ER stress. **(B)** R-Se@MEF2D-siRNA also inhibits cells proliferation by blocking the WNT/β-catenin signaling pathway. **(C)** MEnZn-CuO NPs decreases the expression of HR repairing markers associated with DNA repair mechanisms. **(D)** GNPs reverse EMT by inactivates PI3K/AKT signaling pathway and MAPK signaling pathway.

Apoptosis inhibition is one of the important factors leading to treatment insensitivity and drug resistance in ovarian cancer. In recent years, more and more studies have begun to focus on the promotion of apoptosis through the modulation of apoptosis-related signaling pathways to overcome drug resistance in the non-immune TME. Li et al. (49) designed NPs targeting inhibition of the PI3K/AKT/mTOR pathway to promote apoptosis in AOC cells. They fused cRGD-modified liposomes with CD47-expressing exosomes from TME to form bio-inspired hybrid NPs, and encapsulated triptolide (TP) and miR497 to prepare miR497/TP-HEPs. CD44-expressing exosomes can bind to SIRPα on the surface of tumor cells without being affected by the tumor clearance system, TP and miR497 inhibit the mTOR signaling pathway, reduce cell metabolism to provide energy and nutrients for tumor cells, and make ovarian cancer cells more sensitive to cisplatin treatment. The experimental results show that the PI3K/AKT/mTOR signaling pathway is inhibited by miR497/TP-HENPs-treated ovarian cancer cells, which reduces the expression of anti-apoptotic proteins, such as Bcl-2 and Bcl-XL, promotes apoptosis and overcomes cell drug resistance. In addition, the preparation of NPs that can activate the caspase-mediated apoptotic pathway can also play an equal role in TME. For example, Zhang et al. (59) synthesized silver nanoparticles (AgNPs) with cytotoxicity using a novel bacterium named Bacillus clausii and selected salinomycin (Sal) with cancer stem cell destruction function. The combination of Sal and AgNPs was found to activate caspase-3 and caspase-9, inducing caspase cascade pathways and ultimately leading to apoptosis. Therefore, apoptosis can be induced by NPs modulation of apoptosis-related pathways, thereby overcoming drug resistance in AOC (**Figure 4A**, a).

There are many ways to induce apoptosis, but most current research still focuses on exploring through a single pathway. Although this single-mechanism approach is helpful for understanding the role of specific pathways, its therapeutic effect may be limited by factors such as the complexity of the TME and the drug resistance of tumor cells. Because of this, the construction of a platform for multi-mechanism induction of apoptosis with the help of NPs may provide a more efficient and targeted solution. For example, Qiu et al. (61) devised an amorphous calcium phosphate (CaPO) modified with doxorubicin (DOX)-loaded Arg-Gly-Asp (RGD) (RGD-CaPO/DOX) to induce apoptosis. When absorbed by cells, the NPs release calcium ions and DOX, which is a chemotherapeutic agent that can increase ROS levels to induce ERs. The high concentrations of ROS disrupt the function of calcium channels and calcium pumps on the cell membrane, resulting in increased calcium ion inflow and exacerbating intracellular calcium overload. Their synergistic effect exacerbates mitochondrial dysfunction, which ultimately leads to apoptosis. Furthermore, animal experiments demonstrated that RGD-CaPO/DOX exhibits significant tumor suppression *in vivo*. Tumor cells in mice treated with RGD-CaPO/DOX showed more severe apoptosis than those treated with DOX alone, reflecting the effectiveness and safety of RGD-CaPO/DOX NPs (**Figure 4A**, b).

#### 5.1.2 NPs inhibit the proliferation by regulating the non-immune TME in AOC

As previously described, MEF2D is an important transcription factor in the WNT/β-catenin signaling pathway, leading to tumor proliferation by upregulating the expression of VEGF. Wang et al. (31) attempted to synthesize MEF2D-siRNA-based NPs to achieve effective silencing of MEF2D and thereby inhibit tumor proliferation. They selected selenium nanoparticles (SeNPs) that facilitate drug or gene delivery, installed charged RGDfC peptides on the surface of NPs to enhance targeting, and finally successfully prepared R-Se@MEF2D-siRNA NPs. The results showed that the MEF2D gene in ovarian cancer cells treated with R-Se@MEF2D-siRNA was inhibited, blocking the WNT/β-catenin pathway, thereby reducing VEGF secretion in the non-immune TME, reducing vascularization, thereby reducing nutrient and oxygen supply to tumors, and inhibiting AOC cell proliferation (**Figure 4B**). Therefore, R-Se@MEF2D-siRNA exhibits excellent anti-tumor effects *in vivo* by inhibiting tumor cell proliferation.

#### 5.1.3 NPs prevent DDR by regulating the non-immune TME in AOC

The DDR regulated by the non-immune TME seriously affects the sensitivity of AOC treatment (63). Yi et al. (60) discovered that micelle-encapsulated zinc-doped copper oxide nanoparticles (MEnZn-CuO NPs) can reverse the resistance of ovarian cancer to the PARP inhibitor Olaparib by disrupting homologous recombination (HR) repair. The results showed that MEnZn-CuO NPs significantly reduced the expression of HR repair pathway-related genes (BRCA1, BRCA2, ATM, and RAD51) (**Figure 4C**). Further studies into the effects of MEnZn-CuO NPs have found that they can be used in combination with PARP inhibitors and have shown synergistic inhibition of ovarian cancer cell growth in both *in vitro* and *in vivo* experiments. Therefore, MEnZn-CuO NPs, as a novel nanocomposite, significantly enhance the sensitivity of ovarian cancer cells to the PARP inhibitor by destroying the HR repair ability and have potential application value in reversing drug resistance in the non-immune TME.

### 5.2 NPs reduce tumor metastasis by regulating the non-immune TME in AOC

As previously mentioned, the occurrence of EMT is closely related to the activation of the PI3K/AKT signaling pathway by factors, such as cytokines and growth factors secreted by cancer-associated fibroblast (CAF) and endothelial cell (EC) communication in the non-immune TME. Recently, Zhang et al. (58) prepared 20 nm gold nanoparticles (GNPs) by citrate reduction and found that GNPs down-regulate the expression of key cytokines and growth factors that activate the PI3K/AKT signaling pathway by blocking multicellular communication in the non-immune TME (**Figure 4D**). This down-regulation inhibits the activation of the PI3K/AKT signaling pathway, thereby reversing the EMT process in AOC and reducing the migration and invasion ability of tumor cells. Therefore, the construction of NPs inhibits EMT-related pathways and reduces tumor cell metastasis in non-immune TME, providing a new idea for the treatment of AOC.

## 6 Conclusion

Nanotechnology is a science and technology based on many modern advanced scientific technologies, and it is a product of the combination of dynamic science and modern technology. The latest advances in nanotechnology have accelerated research on NPs construction, making it possible to develop complex drug delivery systems (64). It can enhance the specificity of the drug’s action and reduce toxic side effects on normal organ tissues by integrating multiple drugs together and targeting specific locations in the body to construct NPs. Therefore, NPs have been widely used in the diagnosis and treatment of diseases, especially in the field of cancer. Ovarian cancer is a common malignant tumor in women, and multiple studies have shown that its high mortality and recurrence rates may be related to the regulation of TME. In the TME, tumor cells can alter and maintain their own survival and development conditions through autocrine and paracrine pathways, promoting tumor growth and progression. Whole body and local tissues can also utilize the TME to restrict and influence the occurrence and development of tumors through metabolic and functional changes. Therefore, the complex mechanisms regulated by TME may affect the effectiveness of its adjuvant therapy, and even lead to resistance to chemo-immunotherapy (65). In this review, we elucidate the molecular mechanisms and intervention strategies of TME in regulating the occurrence and development of AOC. In the immune TME, functional inhibition of immune effector cells such as T cells and DCs, as well as abnormal activation of immune suppressive cells such as M2-TAMs, MDSCs, and Tregs, may be closely related to ovarian cancer progression and tumor cell drug resistance. In addition, the non-immune TME regulates abnormal proliferation and migration of ovarian cancer cells, abnormal aerobic glycolysis, apoptosis inhibition, and DDR, leading to poor clinical chemo-immunotherapy efficacy and tumor cell resistance. Reversing drug resistance in ovarian cancer cells and avoiding the recurrence of ovarian cancer is currently a clinical challenge. Many studies have shown that we can construct NPs to activate immune effector cells or block immune suppressive cell activity by regulating TME, enhancing the body’s immune capacity, may achieving clinical effects of reversing drug resistance in ovarian cancer cells and intervening in the development of ovarian cancer (66). We can also regulate the non-immune TME by constructing NPs, promote tumor cell apoptosis, disrupt HR repair, and intervene in the mechanism of EMT occurrence, thereby increasing the sensitivity of ovarian cancer cells to chemo-immunotherapy and intervening in the progression of ovarian cancer. In summary, utilizing nanotechnology to regulate TME may provide a novel therapeutic strategy and approach for AOC.

## 7 Future perspective

The molecular mechanism of TME regulating AOC is still being continuously improved and updated. The application of nanotechnology will bring more hope and expectations in ovarian cancer TME for clinical treatment. PANoptosis is currently a hot topic in the field of cancer research. Although the molecular mechanism of PANoptosis is not yet perfect, utilizing non-immune TME to regulate the PANoptosis effect of ovarian cancer cells may provide additional insights and surprises for the treatment of AOC in the future. PANoptosis is a recently discovered form of cell death that involves the simultaneous activation of multiple cell death pathways, including apoptosis, necrosis, and pyroptosis (67). It has been confirmed that PANoptosis can regulate the occurrence and development of tumors, reversing the drug resistance of tumor cells to chemo-immunotherapy (68). Given that TME may protect ovarian cancer cells from the PANoptosis effect caused by chemo-immunotherapy, we may be able to utilize nanotechnology to construct NPs that regulate TME to enhance the PANoptosis effect of ovarian cancer cells, thereby introducing new targeted and personalized treatment options for AOC patients. Given that the TME can protect tumor cells from killing by PANoptosis, in the near future, we may be able to enhance the sensitivity of ovarian cancer cells to PANoptosis by constructing NPs loaded with regulating TME drugs, thereby introducing new targeted and personalized treatment protocols for patients with AOC.

Due to recurrence and metastasis of AOC, it may be the main research direction for utilizing nanotechnology to regulate the immune TME of ovarian cancer cells and improve clinical adjuvant therapy efficacy in the future. Recent studies have shown that T cells in TME exhibit terminal differentiation depletion under continuous tumor antigen stimulation, commonly referred to as exhausted T cells (Tex cells) (69). It can significantly reduce the self-renewal and effector molecule release ability of Tex cells for a large amount of lactate produced and released by tumor cell metabolism, forming immunosuppressive TME. However, reports have shown that monocarboxylate transporter 11 (MCT11) may be closely related to the absorption of lactate by Tex cells. If specific MCT11 inhibitors can be developed in the future, nanotechnology can be used to construct NPs loaded with MCT11 inhibitors to reverse the immunosuppressive TME of AOC, providing a new concept for adjuvant therapy of ovarian cancer.

The complexity of TME has always been a major obstacle to the clinical adjuvant therapy of AOC. It can provide scientific basis about the application of nanotechnology to regulate TME for the development of new treatment strategies, and promote the personalized and precise development of AOC treatment.

## Glossary

AOC: Advanced ovarian cancer
TME: tumor microenvironment
DDR: DNA damage response
EMT: epithelial-mesenchymal transition
NPs: nanoparticles
ECM: extracellular matrix
ICCs: immunocompetent cells
DCs: dendritic cells
NK cells: natural killer cells
TAMs: tumor-associated macrophages
Tregs: regulatory T cells
MDSCs: myeloid-derived suppressor cells
PD-L1: Programmed cell death ligand 1
STAT3: signal transducer and activator of transcription 3
JAK: Janus kinase
TI-DCs: Tumor-infiltrating dendritic cells
ROS: reactive oxygen species
4-HNE: 4-hydroxynonenal
ER: endoplasmic reticulum
IRE1α: inositol-requiring kinase 1α
XBP1: X-box binding protein 1
FAO: fatty acid oxidation
IL-10: interleukin 10
TGF-β: transforming growth factor β
TCF/LEF: T cell factor/lymphoid enhancer factor
CXCL2: CXC motif chemokine ligand 2
TLRs: Toll-like receptors
TNFRs: tumor necrosis factor receptors
CTLs: cytotoxic T lymphocytes
RIPK1: receptor-interacting serine/threonine-protein kinase 1
IKK: IkB kinase
CTLA-4: Cytotoxic T lymphocyte associate protein-4
Mcl-1: myeloid leukemia factor 1
MCT: monocarboxylate transporter
PDK1: pyruvate dehydrogenase kinase 1
PIP3: phosphatidylinositol triphosphate
GSK3b: glycogen synthase kinase 3b
p70S6K: p70 ribosomal protein S6 kinase
CTR1: copper transporter 1
UBR5: Ubiquitin protein ligase E3 component n-recognin 5
MSV: multistage vector
VSSP: Very small size particles
NAcGM3: N-acetylneuramic acid
OMV: outer membrane vesicles
RSQ: Resiquimod
KT-NE: KIRA6 loaded α-Tocopherol nanoemulsion
PLGA: poly lactic-co-glycolic acid
CpG-ODN: CpG-oligodeoxynucleotide
PFH: perfluorohexane
IRM: hybrid biomimetic coating
IVT: *in vitro*-transcribed
MVB: multivesicular body
GNP: Gold nanoparticle
MEF2D: Myocyte Enhancer Factor 2D
Sal: salinomycin
TP: triptolide
MEnZn-CuO: Micelle Encapsulation Zinc-doped copper oxide
RGD: Arg-Gly-Asp
DOX: doxorubicin
IDEM: immune-derived exosome simulator.
